# The Pre-Medical Health Coach (PHC) program: pre-medical students as volunteer health coaches at a safety-net hospital in California, 2016–2020

**DOI:** 10.1186/s12909-024-06524-6

**Published:** 2025-02-27

**Authors:** Emma B. Shak, Lyn Berry, Bryant Chow, Sharone Abramowitz, Kala M. Mehta

**Affiliations:** 1 Department of Medicine, UC San Francisco, Division of General Internal Medicine, San Francisco Department of Medical Affairs, 401 3rd Street, San Francisco, CA 94107 USA; 2 Department of Medicine, Highland Hospital, Alameda Health System, (emeritus) 1411 E. 31st Street, Oakland, CA 94602 USA; 3 College of Osteopathic Medicine, Touro University, 1310 Club Dr, Vallejo, CA 94592 USA; 4 Department of Epidemiology and Biostatistics, University of California San Francisco, San Francisco, CA 94143 USA

**Keywords:** Race, Ethnicity, Underrepresented in medicine, Health professions, Health coach, Pre-medical student, Chronic disease self-management

## Abstract

**Background:**

The volunteer Pre-Medical Health Coach (PHC) program engages student volunteers, in team-based primary care providing self-management support to patients with chronic conditions. Both the PHCs and the patients they serve are diverse. The aims of this study are to assess the impact of the PHC program on student outcomes and patient biomarkers.

**Methods:**

All PHCs were students recruited from local universities, interviewed, then trained in motivational interviewing and evidence-based chronic disease self-management support. The 22 PHCs were diverse – 8 (36.3%) were underrepresented in medicine and 2 (9%) were first generation in college. The study setting was a public safety-net adult medicine outpatient clinic in Oakland, California. PHCs spent 5 h weekly, for 1–3 years, volunteering alongside primary care residents and faculty. Duties included: (1) observing the doctor-patient interview, (2) on-site health coaching, and (3) telephone follow-up. An internal medicine physician and a licensed clinical social worker supervised the program. Surveys were implemented to understand program impacts and career trajectories of the 22 PHCs and health action plans were reviewed over 4 years. In addition, a focus group was conducted with the PHCs using a deductive approach.

**Results:**

Two years after completing the program, 90.9% of the PHCs were still on the path to health professions programs, 50% had applied to medical school, and 18.2% started medical training. Qualitative impacts on coaches included significant clinical exposure, meaningful connection with patients, ‘bridging’ between the community and health care system and having a more holistic view on health. Patient biomarkers included a decrease in hemoglobin A1c level by 1% point in diabetic patients with diabetes-focused action plans, and smoking-focused action plans led to decreased smoking or cessation.

**Conclusions:**

The Pre-Medical Health Coach (PHC) program may benefit both PHCs and patients. PHCs were diverse and providing chronic disease self-management support to patients in a safety-net health care system. This program could be readily adapted in health care systems nationwide to increase diverse entrants to the healthcare workforce.

**Supplementary Information:**

The online version contains supplementary material available at 10.1186/s12909-024-06524-6.

## Background

Evidence-based health coaching models are widely successful and address a variety of chronic disease conditions managed in primary care [1–3]. In some settings, health coach interventions are associated with improved biomarkers, like hemoglobin A1c or lipid levels [1, 4]. Although these models work for patients in a range of clinical settings, individual behavior change remains challenging, particularly in safety-net settings [1, 5]. Primary care providers (PCPs) often are not able to dedicate extensive individualized attention during and after a clinic visit, and may become frustrated when patients fail to follow-up with improved self-care [6, 7]. One strategy for addressing these challenges in the safety-net is to enhance the primary care team, at the point of care, through the addition of health coaching using the ‘teamlet’ model, wherein dedicated health coaches work side-by-side with PCPs [8, 9].

One key feasibility challenge to implementing effective health coaching in primary care is identifying and training appropriate health coaches. In the United States, our healthcare workforce does not adequately reflect the linguistic and ethnic diversity present in many urban populations [10]. Pre-medical students, particularly those who are from diverse groups [11], have the cultural wealth of their communities and at the same time may not have an easy entry point to experience the range of medical careers, or may have inadequate guidance during their educational career [12]. Though several pathway programs exist to increase diversity in the health professions nationwide, none use health coaching. By linking diverse pre-medical students to health coaching positions, and having coaches from different backgrounds mix, the students both can learn from each other and gain exposure to clinical settings. This experience could guide them towards their career goals as health professionals, especially towards primary care, where there is a workforce shortage. In addition, having health coaches in primary care extends the capacity of overstretched clinicians and medical systems, which is of particular importance within the safety-net.

Therefore in 2008, members of our Primary Care Division instituted a pre-medical student health coach program for team-based primary care at an urban safety net hospital in California. This Pre-Medical Health Coach (PHC) program used a motivational interviewing-based, chronic care model curriculum for training health coaches and ensuring the quality of care they delivered [13, 14]. To our knowledge, this was the first health coach program to engage volunteer pre-medical students as coaches; it was first described in 2010 [15].

## Methods

### Setting and participants

The setting for this program is the Adult Medicine Clinic at Highland Hospital, within the Alameda Health System (AHS) in Oakland, California. Highland is a public safety net hospital serving a culturally diverse and predominantly low-income Medicaid and uninsured patient population within Alameda County. Although this project began in 2008, the current review encompasses the years 2016–2020, during which 22 pre-medical students participated.

#### Ethics approval and consent to participate

The AHS Institutional Review Board reviewed and approved a verbal consent procedure for this study, and authors report no conflicts of interest.

### Program description

#### Recruitment

Using email announcements, leaders of the program recruited student volunteers from local post-baccalaureate and undergraduate pre- medical programs. Pre-requisites included status as a senior or beyond in their undergraduate studies, ability to commit to participation for a full academic year, and ideally bilingual in the most common non-English languages represented in our clinic. After submitting a written personal statement and participating in an interview, candidates were selected by the program director (EBS) based on these criteria. Eight health coaches per year were accepted into the program.

#### Training

Preceding the on-site orientation, PHC training consisted of approximately 4 h of self-study: reviewing a compilation of community resources, watching on-line motivational interviewing training videos, and reading patient education handouts about common primary care conditions [16–25]. This was followed by 8 h of interactive on-site training, which included role plays, motivational interviewing, overview of substance use disorders, and basics of nutrition, diabetes, and hypertension.

#### Clinic integration and workflow

**Fig. 1:**
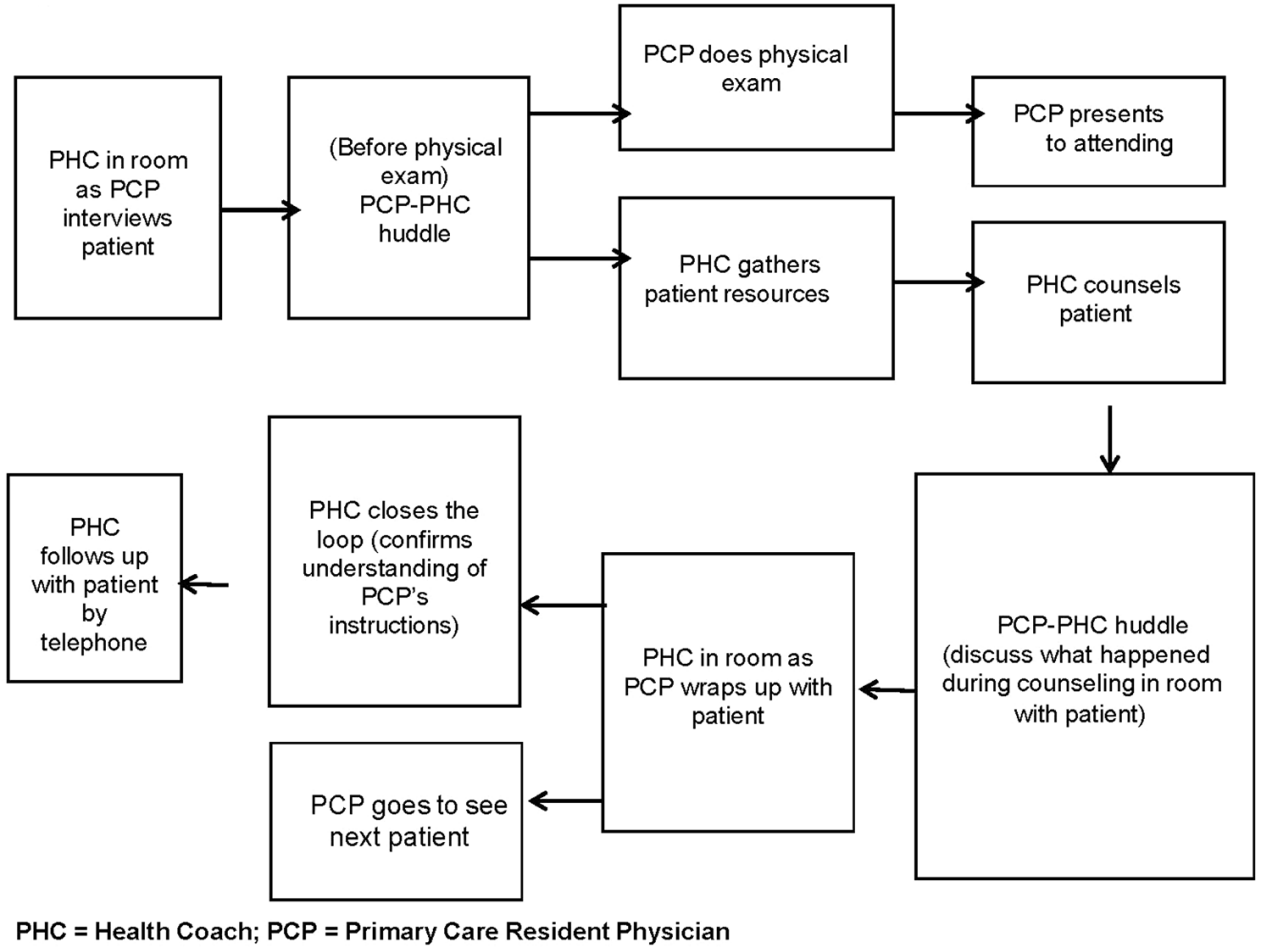
Workflow for Pre-Medical Health Coach Program, Alameda Health System. PHC = Health Coach; PCP = Primary Care Resident

PHCs were on site in clinic for one half-day per week (5 h) and were assigned to a practice teamlet with 2–3 internal medicine residents per year and/or 1–2 primary care faculty members. We had two health coaches simultaneously in clinic working with two different teams so that the PHCs could serve as resources for one another. The PHC program was introduced to the residents during intern orientation and reintroduced to senior residents during didactics sessions. Clinic staff were introduced to new health coaches and to the program overall at monthly team meetings and daily morning huddles. In brief, the PHC workflow (Fig. 1) included: the coaches observe the doctor-patient interview, then provide on-site health coaching during resident case presentation, and later perform patient telephone follow-up as needed. The content of health coaching sessions (both in-person and telephone follow-up), including the patient’s specific action plan, was documented in the electronic health record as a Health Coach Note and on separate forms that the health coaches used to track their caseload.

#### Physician supervision

Ongoing supervision was conducted through weekly group debriefing of the health coach’s cases, either with the project supervisor or experienced peer health coaches. These debriefing sessions occurred every day that coaches were in clinic, from 12:30 − 1:00 PM, the time when the morning and afternoon PHC’s schedules overlap. In addition, 1–2 in-service sessions per semester were conducted in lieu of a debriefing session to review counseling techniques and introduce new didactic content. A supervisor reviewed all PHC notes entered into the medical record for the first few months of the academic year, giving feedback as needed. In summary, 14 h of faculty time were dedicated to recruitment, six hours for additional MI training in the Spring and faculty contributed 30 min per week for case supervision for 10 months.

#### Model fidelity

The PHC program used a health coaching approach based on self-management support (SMS) principles, as defined by the Chronic Care Model (CCM) and articulated by Bodenheimer et al. “Self-management support” is “the systematic provision of education and supportive interventions by health care staff to increase patients’ skills and confidence in managing their health problems, including regular assessment of progress and problems, goal setting, and problem-solving support”. The chronic care model outlines key elements of a health system that promotes high-quality chronic disease care; the community, the health system, self-management support, delivery system design, decision support and clinical information systems [15, 17–19, 26, 27].

Brief Motivational Interviewing (MI) [2, 13, 14], a patient- centered counseling style for addressing ambivalence about change, was the backbone of the training for premedical health coaches [2, 20]. MI was used because it has a substantial evidence base, has been adapted into a brief primary care version, and because professional degree status does not influence quality, nor do providers’ baseline characteristics. This integrated approach of MI, the CCM, and Prochaska’s Stages of Change model echoes the curriculum of the primary care internal medicine residency program at Highland Hospital [20].

### Program evaluation

Anonymous questionnaires were used to assess the impact of this volunteer experience on PHCs’ learner outcomes. These surveys were administered yearly in the late spring using RedCap.

A focus group was conducted to address the same domains as the surveys, data collection and thematic analyses were informed by instructivist principles. All current PHCs were invited to participate in the focus group and 6/8 participated. The focus groups lasted 1.5 h and the discussions structured by an interview guide and was conducted by a researcher trained in qualitative methodology. Discussions were recorded and transcribed, redacted, and coded using Dedoose software (Dedoose.com, Los Angeles, US). Applying a deductive approach, the focus group transcript was analysed using open coding by two members of the research team (KM and ES). Preliminary codes were cross-checked and discrepancies discussed until consensus was reached, but inter-coder reliability was not quantified. The codes were organized into themes and relationships.

Patient level demographic characteristics and biomarkers were extracted from a paper-based action plan. Data from action plans were added to the REDCAP database without identifiers. No patient level identifiers were added to the database so confidentiality could be maintained. Patient level biomarkers were assessed in 101 patients seen by these coaches over the course of 4 years. The only health metrics were those recorded in the action plans. Inclusion criteria were that these patients were all seen by a PHC. No other inclusion or exclusion criteria were applied. All statistical analyses were conducted using STATA16 College Station, TX: StataCorp LP.

## Results

PHCs median age was 24.5 years old, and they were more often women (14,63.6%). Over one third (8, 36.3%) were from groups underrepresented in medicine (Table 1). According to the US Census Bureau, the Alameda County demographics in 2020 were similar to that of the health coaches (Table 1): 30% white (non-Hispanic), 10.2% black or African America, 32.3% Asian, and 22.2% Hispanic or Latino. On average, the health coaches spent about a year in the health coaching program (1.14 years, SD 0.35).

**Table 1:** Pre-Medical Health Coach characteristics at Alameda Health System from 2016–2020 (*n* = 22)

Factor	Level	*N* (%)
Age, median (IQR)		24.5 (23.0, 25.0)
Sex	Women	14 (63.6%)
	Men	8 (36.3%)
Underrepresented in Medicine		8 (36.3%)
Race/Ethnicity	African American	1 (4.5%)
	Latine	2 (9.09%)
	Filipine	2 (9.09%)
	Vietnamese	2 (9.0910%)
	Caucasian, Filipino, Japanese	1 (4.5%)
	Chinese	1 (4.5%)
	Asian Indian	3 (13.6%)
	Middle Eastern	1 (4.5%)
	White	8 (36.3%)
	Unreported	1 (4.5%)
First Generation	Yes	2 (9.09%)
Still in science	Yes	20 (90.9%)
Completed a Prep Program	Yes	4 (18.18%)
Graduation	Completed Undergrad	18 (81.81%)
Applying to medical school, Current Cycle		8 (36.3%)
Applying to medical school, Next Cycle		3 (13.63%)
	Physician Assistant program	1 (4.5%)
	MS1	2 (9.09%)
	MS2	1 (4.5%)
	MS3	1 (4.5%)
	PhD/PsyD program	2 (9.09%)%)
	Unreported	1 (4.5%)
Mean ears as a Coach (SD; range)		1.14 (0.35; 1–3)
Average time from patient first seen by coach to last follow up (Mean in days, SD)		43.2 (90.8)

Preliminary results show the positive learner impact of Pre-Medical Health Coaching in residency clinics. As of 2020, 20 participants (90.9%) continued in science, 18 (81.81%) had completed their undergraduate education, 11 (50%) were in the process of applying to medical school, and 4 (18.18%) had entered medical training. Two people chose medicine-adjacent PhD careers (9.09%) and one PHC chose not to report this information (4.5%).

Qualitative impacts on PHCs fell into the following themes: significant clinical exposure and experience, an ability to overcome career barriers, a sense of community and value to patients, and having a more holistic view on health. One PHC stated that “We got to actually form a relationship with [the patients] and follow up with them which I thought was really unique.” Another stated that “This position was more…active…Maybe shadowing or, for example, scribing, could be passive.” And the community impact is exemplified by the following quote: “I’m from [Oakland], born and raised… It was a great opportunity and incredible just for me to come back and be part of… a team that’s helping people in my community” (see Table 2).

**Table 2:** Domains and relevant themes derived using grounded theory [35] from focus group with Pre-medical Health Coaches at Alameda Health System in 2020 (*n* = 6)

Domain	Quotation
Career impact of pre- medical health coach	“[During the application process, ] we want to be able to say that we had experience with a patient, we talked to them, we impacted them in some way… in the health coach position… they all do in the long haul.” “We got to actually form a relationship with [the patients] and follow up with them which I thought was really unique… in this program that we actually followed up with them over… weeks or months.” “[We were] able to see the role of what the doctors are doing and the passion that they have when working with their patients. They go way beyond what you would imagine anybody would do for their patients, so seeing that love for it also kind of strengthened my interest in this field [of primary care], definitely.”
Personal impact	“I think it was really valuable to be able to have a more long-term perspective around patient care.” “I was very aware that in this transition to pre-med stuff, there would be a hole in my life. I wasn’t actually of use beyond developing my own brain. Health coaching was like the most fulfilling thing I’ve done this year. No question.”
Connection to patients	“In the emergency department for example, a lot of times you don’t get that close contact with either the patients or the healthcare professionals, as volunteers. Being able to [be]
	the provider at that moment with the patient when it’s me and them – that connection is unbeatable.” “We take the small steps to allow them to be able to achieve [their goals] later on. We don’t expect them to meet those goals right away but we identify what we can do that day.”
Overcoming career barriers	“This position was more… active. Usually, if I was doing other pre-med clinical activities, it’s really passive. Maybe shadowing or, for example, scribing could be passive.” “It definitely strengthened my intent to pursue a career, just by seeing that structure [of medical training]. Also, just… observing… how residents are with their patients, how residents are with their attendings, and things like that.” “…my relationships with the rest of… the providers would have been different if I was just shadowing. Like, we were bringing something to the table and that was important for them investing in us [health coaches]. It wasn’t a one-way relationship always.”
Community/value to patients	“I’m from [Oakland], born and raised. So… [Highland Hospital] has always been a big part of, you know, growing up … Everybody knows [Highland]. It was a great opportunity and incredible just for me to come back and be part of… a team that’s helping people in my community.”
	“As part of our health coaching program we provide a lot of community resources and things like that. I learned about all the different types of programs, all of these resources for patients and staff. I feel like that was really cool to learn about.”
Medical/layperson bridge	“But there were some patients where, …when the provider was in the room, it almost seemed like they wanted to… please them and just like nod along. But then… once the provider left was like, ‘okay, now let’s get into it.’ I don’t want [say] ‘oh, health coaches understand patients better all of the time.’ That’s not what it is at all. For some patients, I think it really was useful to just have someone that they were able to see as more on their level.”
Holistic view of health	“There were a couple cases where…[with] the quick time with the patient, there was [focus on] physical exam and things like that. But not understanding that [the patient was] was struggling with depression and should be handed off to behavioral health. Only after the health coach conversation, …so then I would leave the room and talk to the resident and say here’s what’s happening. So please go tap in behavioral health. [The patient] got a service they wouldn’t have gotten otherwise.” “I found that you really get a sense for all the upstream effects… on people’s health. Like when they come into the clinic, a lot of people are homeless or one thing or another has happened to them. I think the clinic [is] an environment where the other providers are really aware of that kind of thing. Like what people are eating at home or do they have a home or these kinds of things. Which was like not always the easiest lesson to digest, I think, as somebody who’s thinking about becoming a provider in this kind of field.”

There were 426 patients seen by PHCs over the course of the study, of whom 234 had at least one follow-up visit and 101 had data on the condition identified on their action plan, such as weight, hemoglobin A1c, or smoking (Table 3). The 234 patients who participated in follow-up from a health coach were similar to those not seen for follow-up. Patients were on average 56 years old, more often women, and spoke a variety of languages (listed in Table 3), as is representative of the Alameda Health System population. Diabetes and hypertension were the most common primary diagnoses of the patients seen by PHCs.

**Table 3:** Patient sociodemographic characteristics according to action plan target (*n* = 101)

		Weight	Diabetes	Smoking	Total
Factor		Level	(N, %)	(N, %)	(N, %)
N		75	18	8	101
Age, mean (SD)		57.45(47.3)	49.9(10.7)	58.28 (13.6)	56.0(40.1)
Sex	Female	47, 62.7%	8, 44.4%	3, 37.5%	58, 57%
	Male	28, 37.3%	10, 55.6%	5, 62.5%	43, 43%
Language	Amharic	1, 1.3%			1, 0.9%
	English	30, 40%	4, 22.2%	2, 25%	36, 35.6%
	Portuguese	1, 1.3%			1, 0.9%
	Chinese	1, 1.3%			1, 0.9%
	Spanish	17, 22.7%	7, 38.9%		24, 23.7%
	Tigryna	1, 1.3%			1, 0.9%
	Tagalog	1, 1.3%			1, 0.9%
	Vietnamese	1, 1.3%			1, 0.9%
	Other	22, 29.3%	6, 33.3%	6,75%	35, 34.6%
Insurance Status	Medicare	7, 9.3%	1, 5.6%		8, 7.9%
	Medicaid	30, 40.0%	5, 27.6%		36, 35.6%
	Self Pay	3, 4.0%			3, 2.9%
	Missing	35, 46.7%	12, 66%	7, 87.5%	54, 53.4%

We analyzed data for the three most common action plans and report the corollary biomarkers. Patients with uncontrolled diabetes (hemoglobin A1c > 8.0%) who were seen by a health coach at baseline *and* for at least one follow-up telephone session (*N* = 18) had an A1c 1.14% points lower than those who were not seen for follow-up by a PHC. For patients with a diet or exercise action plan (*N* = 75), we saw no change in BMI. Lastly, for the 8 patients who had a smoking-oriented action plan, daily cigarette use decreased by an average of 6.3 cigarettes.

## Discussion

More than 90% of health coaches remained in science (e.g., as graduate students, health and lab technicians, etc.) and 18% went on to enter medical school. With longer follow up, we hypothesize that the proportion entering will increase. Perhaps most importantly, the program provides a substantive clinical experience that is rare for medical school applicants. Though the number of patients studied was low, and could be considered preliminary, patients who had at least one follow-up with a PHC were found to have an A1c that was 1% point lower than those without PHC support, and daily cigarette use decreased. There was no discernible change in BMI during the time we examined.

Health coaching by volunteers is an established approach for improving patient self-efficacy in chronic disease management and also patient satisfaction [21–25]. To our knowledge, this is the first primary care health coaching model that utilizes unpaid pre-medical student volunteers. Importantly, this program creates advantages for diverse medical school applicants by providing them with critical clinical experience that strengthens their status as applicants to post- graduate programs of any kind.[28–35] By preferentially selecting coaches who reflect the demographics of the community served by Alameda Health System, we can achieve higher rates of racial and cultural concordance between coach and patient. Beyond language concordance, we know that racial and cultural concordance leads to better outcomes for patients and is more patient-centered [36, 37]. We continue to strengthen our recruitment and outreach approaches, and in 2021–2022 and 2022–2023, 50% of the PHCs identify as URiM (4/8) and in 2023–2024 75% identify as URiM (6/8).

Beyond the impact of the PHC model on both learners and patients, there are also benefits to the larger health system of the primary care clinic. Volunteer health coaches become an integral part of the primary care team. Coaches can give motivational interview-based counseling the time it requires and can explore and address psychosocial barriers to chronic disease self-management, thereby also reducing the workloads of other providers. Strengths of this study include the rich qualitative data from health coach interviews, a novel health coaching model that can be adapted to a wide variety of outpatient primary care clinics, and a strategy to tackle underrepresentation in medical education. As this was a retrospective study with a focused qualitative component, without systematic patient data follow-up, limitations include limited patient biomarker data, likely a timeframe too-short to see effects on weight, no data on long term effects, no data since 2020, no patient satisfaction data, no data on satisfaction of other team members, no patient or student comparison group and potential selection bias within the focus group, as well as lack of generalizability given the small sample size of students. Our team plans to undertake a future prospective and qualitative study to address some of these limitations.

We have learned some important lessons from our experience with this model. One critical aspect of successful student health coaching is selecting coaches with the maturity and poise to work directly with patients with minimal supervision. We predominantly chose applicants who were in post-baccalaureate programs, only rarely selecting students in their third or fourth year in college. Also, we relied heavily on in-person interviews when selecting PHCs. Health coach retention was occasionally an issue, as busy students sometimes became overcommitted and unable to adhere to a 5-hour per week volunteer assignment. Of course, more patient contact hours could strengthen the experience for the coaches. The mentoring and training of the health coaches would benefit from a validation process; it also requires significant time investment by the mentor(s)—often busy internal medicine and behavioral health providers—and requires institutional support. Finally, thoughtful planning for integrating the coaches into the clinical team is very important for the program’s success (see Clinic Integration section above). In our experience, clinic staff concerns about how the program might adversely impact clinic flow, space constraints, etc. were quickly mitigated by the value the health coaches brought to the clinic overall. As one health coach noted, “…my relationships with the rest of… the providers would have been different if I was just shadowing. Like, we were bringing something to the table and that was important for them investing in us [health coaches]. It wasn’t a one-way relationship ….” (Supplemental Table 1) [38].

## Conclusions

The Alameda Health System Pre-Medical Health Coach (PHC) program is a novel care delivery model for recruiting and training volunteer pre-medical students, preferentially from diverse backgrounds, to serve as health coaches alongside Internal Medicine residents in an academic primary care clinic. This model could be readily applied across a variety of ambulatory care settings. This model provides meaningful clinical experience for pre-medical students and provides patients with chronic disease self-management support. More data are needed to clarify the long-term impact of this program on biomarkers for patients and on career trajectories of pre-medical students.

## Electronic Supplementary Material

Below is the link to the electronic supplementary material.

Supplementary Material 1

Supplementary Material 2

## Data Availability

The datasets used and/or analysed during the current study available from the corresponding author on reasonable request.

## Declarations

### Permissions

All short quotes from the focus group obtained informed consent and permission to publish, sources were cited in text.

### Previous presentations

E Shak, D Flattery, B Chow, KM Mehta. “The Pre-Medical Health Coach (PHC) Program: Pre-Medical Students as Volunteer Health Coaches at a Safety-Net Hospital in California, 2016–2020.” Poster Presentation at the Society of General Internal Medicine Annual Meeting, April 20–23, 2021.
