# Supplementary material for: The Pre-Medical Health Coach (PHC) program: pre-medical students as volunteer health coaches at a safety-net hospital in California, 2016–2020
Source: BMC Med Educ. 2025 Feb 27;25:322. doi: 10.1186/s12909-024-06524-6 (PMC11869730; doi:10.1186/s12909-024-06524-6)
Supplement: Supplementary file 1 — Supplementary Material 1 [file 12909_2024_6524_MOESM1_ESM.docx]

Supplemental figures

Supplemental Figure 1. Change in Hemoglobin A1C for diabetic patients, change in body mass index (BMI) for patients with self-management action plans addressing diet and exercise, and total cigarettes per day in smoking patients* who were seen for at least one follow-up by premedical health coaches at Alameda Health System (n=101)

Hemoglobin A1C


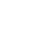

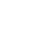


P=0.05

P=0.98

P=0.06**

3.0

Baseline Follow-up Mean Mean

Axis Title

Smoking

9.3

15.0

10.0

5.0

0.0

Baseline Follow-up Mean Mean

Axis Title

36.2

36.2

40.0

30.0

20.0

10.0

0.0

BMI

Follow-up Mean

Baseline Mean

**9.4**

**10.5**

C) Smoking (n=8)*

B) BMI

(n=75)

A) Hemoglobin A1C (n=18)

**p values calculated using paired t-tests for patients with at least one follow-up measurement

Body Mass Index

Average number cigarettes/day

| Supplemental Table 1. Domains and relevant themes derived using grounded theory^35^ from focus group with  Pre-Medical Health Coaches at Alameda Health System in 2020 (n=6) | |
| --- | --- |
| Domain | Quotation |

| Career impact of pre- medical health coach | “[During the application process,] we want to be able to say that we had experience with a patient, we talked to them, we impacted them in some way… in the health coach position… they all do in the long haul.”  “We got to actually form a relationship with [the patients] and follow up with them which I thought was really unique… in this program that we actually followed up with them over… weeks or months.”  “[We were] able to see the role of what the doctors are doing and the passion that they have when working with their patients. They go way beyond what you would imagine anybody would do for their patients, so seeing that love for it also kind of strengthened my interest in this field [of primary care], definitely.” |
| --- | --- |
| Personal impact | “I think it was really valuable to be able to have a more long-term perspective around patient care.”  “I was very aware that in this transition to pre-med stuff, there would be a hole in my life. I wasn't actually of use beyond developing my own brain. Health coaching was like the most fulfilling thing I've done this year. No question.” |
| Connection to  patients | “In the emergency department for example, a lot of times you don't get that close contact  with either the patients or the healthcare professionals, as volunteers. Being able to [be] |

|  | the provider at that moment with the patient when it's me and them – that connection is unbeatable.”  “We take the small steps to allow them to be able to achieve [their goals] later on. We don't expect them to meet those goals right away but we identify what we can do that day.” |
| --- | --- |
| Overcoming career barriers | “This position was more… active. Usually, if I was doing other pre-med clinical activities, it's really passive. Maybe shadowing or, for example, scribing could be passive.”  “It definitely strengthened my intent to pursue a career, just by seeing that structure [of medical training]. Also, just… observing… how residents are with their patients, how residents are with their attendings, and things like that.”  “…my relationships with the rest of… the providers would have been different if I was just shadowing. Like, we were bringing something to the table and that was important for them investing in us [health coaches]. It wasn't a one-way relationship always.” |
| Community/value to patients | “I'm from [Oakland], born and raised. So… [Highland Hospital] has always been a big part of, you know, growing up … Everybody knows [Highland]. It was a great opportunity and incredible just for me to come back and be part of… a team that's helping people in  my community.” |

|  | “As part of our health coaching program we provide a lot of community resources and  things like that. I learned about all the different types of programs, all of these resources for patients and staff. I feel like that was really cool to learn about.” |
| --- | --- |
| Medical/layperson bridge | “But there were some patients where, …when the provider was in the room, it almost seemed like they wanted to… please them and just like nod along. But then… once the provider left was like, ‘okay, now let's get into it.’ I don't want [say] ‘oh, health coaches understand patients better all of the time.’ That's not what it is at all. For some patients, I think it really was useful to just have someone that they were able to see as more on  their level.” |
| Holistic view of health | “There were a couple cases where…[with] the quick time with the patient, there was [focus on] physical exam and things like that. But not understanding that [the patient was] was struggling with depression and should be handed off to behavioral health. Only after the health coach conversation, …so then I would leave the room and talk to the resident and say here's what's happening. So please go tap in behavioral health. [The patient] got a service they wouldn't have gotten otherwise.”  “I found that you really get a sense for all the upstream effects… on people's health. Like when they come into the clinic, a lot of people are homeless or one thing or another has happened to them. I think the clinic [is] an environment where the other providers are really aware of that kind of thing. Like what people are eating at home or do they have a home or these kinds of things. Which was like not always the easiest lesson to digest, I  think, as somebody who's thinking about becoming a provider in this kind of field.” |
