# Supplementary material for: The Pre-Medical Health Coach (PHC) program: pre-medical students as volunteer health coaches at a safety-net hospital in California, 2016–2020
Source: BMC Med Educ. 2025 Feb 27;25:322. doi: 10.1186/s12909-024-06524-6 (PMC11869730; doi:10.1186/s12909-024-06524-6)
Supplement: Supplementary file 2 — Supplementary Material 2 [file 12909_2024_6524_MOESM2_ESM.docx]

Supplementary File 2

Appendix 1. Pre-Health Coach Survey,

Volunteer Health Coach Survey

1. Age
2. Gender Orientation/Sex :
3. Race (list all):
4. Ethnicity
5. What is/was your undergraduate major?
6. Where did you attend undergraduate education/college?
7. Have you completed undergrad?
8. Are you currently in a training program? Which one?
9. Have you taken the MCAT exam? If so, when?
10. Have you already or are you currently applying to medical school (will submit in September, 2019)? Please list date(s) of application.
11. Applying this cycle; submitting in June 2020
12. What schools did you apply to?
13. Have you been accepted to a medical school program? If so, which one? When did you get your acceptance?

Appendix 2. Focus group probes

1. Describe your pre-medical health coaching experience.
2. What were the relationships like with the residents and physicians?
3. How active or passive was your role as a pre-medical health coach?
4. How would you contrast this program to other programs for pre-medical students?
5. What were the differentiators or characteristics of the patients you saw as pre-medical health coaches?
6. Prior to the PHC program, did you experience any barriers to finding pre-medical experiences in a clinical setting? What were the barriers?
7. To what extent were you seen as part of a team?
8. How did the patients view your role as a Pre-medical health coach?
9. How did your experience with premedical health coaching impact your future career goals or aspirations?
10. Is there anything else we missed or you would like to add about the pre-medical health coach program?
